# Effects of stachydrine on norepinephrine-induced neonatal rat cardiac myocytes hypertrophy and intracellular calcium transients

**DOI:** 10.1186/1472-6882-14-474

**Published:** 2014-12-08

**Authors:** Chen Zhang, Xiao-Li Shan, Yue-Ling Liao, Pei Zhao, Wei Guo, Hong-Chang Wei, Rong Lu

**Affiliations:** Department of Pathology, Shanghai University of Traditional Chinese Medicine, Shanghai, 201203 China

**Keywords:** Ca^2+^ transients, Cardiac hypertrophy, Phospholamban, SERCA2a, Stachydrine

## Abstract

**Background:**

*Leonurus heterophyllus* sweet has been suggested to have cardioprotective effects against heart diseases, including ischemic diseases and ventricular remodeling. However, the active ingredients of the herb and the underlying mechanisms are poorly understood. The aim of the present study was to investigate the effects of stachydrine (STA), a major constituent of *Leonurus heterophyllus* sweet, on norepinephrine (NE) induced hypertrophy and the changes of calcium transients in neonatal rat cardiomyocytes.

**Methods:**

Ventricular myocytes from 1-day-old Wistar rats were isolated and cultured in DMEM/F12 with 1 μmol/L norepinephrine in the presence or absence of 10 μmol/L STA for 72 h. Cardiomyocytes hypertrophy was evaluated by cell surface area, total protein/DNA content, β/α-MHC mRNA ratio. While calcium handling function was evaluated by Ca^2+^-transient amplitude and decay, SERCA2a activity and expression, PLN expression and phosphorylation. β1-adrenergic receptor system activation was evaluated by the content of cAMP and the activation of PKA.

**Results:**

NE treatment increases the cell surface area, protein synthesis, the expression level of β-MHC and β/α-MHC ratio. These effects were attenuated by STA. NE-induced hypertrophy was associated with increased Ca^2+^-transient amplitude, accelerated decay of the Ca^2+^-transient, increased phospholamban expression, hyper-phosphorylation at both the serine-16 and threonine-17 residues, increased intracellular cAMP level, and PKA overactivation. All of which were significantly inhibited by STA.

**Conclusion:**

These data suggest that STA attenuates norepinephrine-induced cardiomyocyte hypertrophy and has potential protective effects against β-adrenergic receptor induced Ca^2+^ mishandling.

## Background

Cardiac hypertrophy, which is characterized by increased cell size, protein synthesis and reactivation of the fetal gene program, is an adaptive physiological process in response to various extracellular stimuli [1].

Elevated activities of the adrenergic nervous system (ANS) play central roles in heart failure. The ANS neurotransmitter norepinephrine (NE) mediates effects in cardiac myocytes by binding to adrenergic receptors. β1-AR is the predominant subtype in the myocardium, representing 75% to 80% of total β-AR. β1-AR induces the formation of cAMP and subsequent activation of PKA. PKA in turn phosphorylates several downstream targets which are the key players in cellular calcium handling. Those targets include: L-type Ca^2+^ channel, Ryanodine receptor (RyR) and phospholamban (PLN) [2, 3]. However, β-AR singaling pathoways undergo a number of adaptive and maladaptive regulatory changes as a consequence of heart failure. Chronic increasing of circulating catecholamine causes desensitization and downregulation of cardiac β-AR. Moreover, the β-AR blocker therapy improves survival in patients with chronic heart failure which is strongly suggestive that β-AR hyper-reactivation is involved in heart failure progression [4].

*Leonurus heterophyllus* Sweet, commonly called motherwort, belongs to the Labiatae family, is a traditional Chinese medicine. The herb, named “Yi Mu Cao” in Chinese, or “mother-benefiting herb” if translated literally, has been used traditionally in gynecology and obstetrics. During the last few decades, experimental studies and clinical trials have demonstrated that the herb has pharmacological effects on ischemic diseases by increasing coronary blood flow, improving heart function, and inhibiting blood platelet aggregation [5]. Recently, our group determined that aqueous extracts and alkaloids extracted from *Leonurus Heterophyllus* Sweet have beneficial effects on left ventricular dysfunction or remodeling in rats [6, 7]. However, the active ingredient of the herb and the underlying mechanisms are unknown.

In the present study, we examined the effects of the crystalline alkaloid STA, which is one of the major constituents of *Leonurus heterophyllus* Sweet, on norepinephrine-induced neonatal rat cardiomyocyte hypertrophy and SR Ca^2+^ handling.

## Methods

This study was approved by the Institutional Animal Care and Use Committee of Shanghai University of Traditional Chinese Medicine.

### Neonatal cardiac myocyte culture and treatment protocol

Cardiac myocytes were obtained from the ventricles of 1-day-old Wistar rats and prepared using a modified method as described previously [8]. The cardiac myocytes were plated in DMEM/F-12 (1:1) with 10% horse serum and 5% fetal calf serum. After 48 hours, the cardiac myocytes were incubated in serum-free DMEM/F12 supplemented with 1 ug/mL insulin, 5 μg/mL transferrin and 0.1 mmol/L BrdU. The cardiac myocytes were then treated with norepinephrine (NE, 1 μmol/L) for 72 hours in the presence or absence of carvedilol (CAR, as a positive control, 1 μmol/L) or STA (10 μmol/L), whereas the controls were cultured in medium without treatment.

### Chemicals and reagents

**Figure 1:**
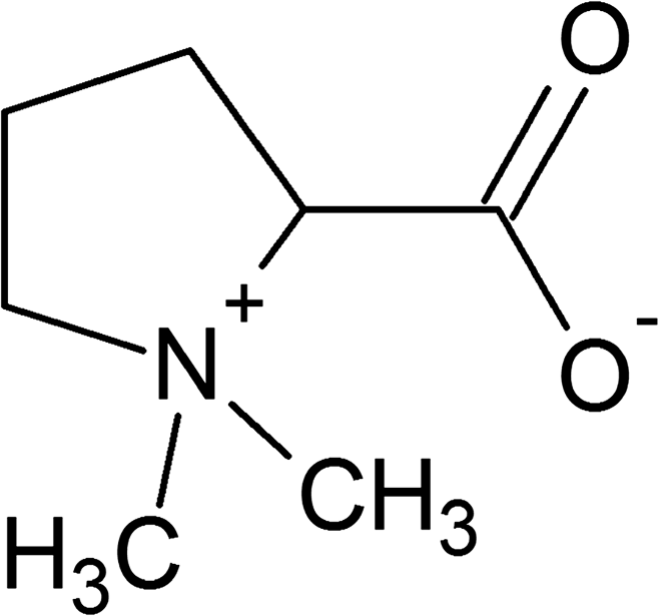
**Chemical structure of stachydrine.**

Chemically pure STA was obtained from the chemistry laboratory at the Institute of Chinese Materia Medica, Shanghai University of Traditional Chinese Medicine. It was identified on the basis of chemical and spectroscopic evidence using the general formula C_7_H_13_NO_2_. The structure of STA is illustrated in Figure 1[9]. Carvedilol was purchased from the Boehringer Mannheim Company. All chemicals were of analytical grade or the best grade commercially available.

### Cell surface area analysis

The cardiac myocytes were washed in Ca^2+^- and Mg^2+^-free PBS and fixed in 3.7% paraformaldehyde-PBS (Fisher, Fairlawn, NJ, USA) for 10 minutes at room temperature, then washed in 0.1% Triton X-100 for 5 min. The cells were double-labeled with DAPI (for DNA nuclear labeling) and phalloidin (for F-actin labeling) conjugated with Alexa Fluor 488 (Invitrogen, Carlsbad, CA, USA). Fluorescently labeled cells were then viewed using a fluorescent microscope (Axiovert 40 CFL, Carl Zeiss, Germany). The cells within each field were outlined, and the two-dimensional area was obtained using I-solution software (IMT i-Solution Inc., Daejeon, Korea). The final mean surface area of each group was determined using 150 cells from 3 independent experiments.

### Total protein/DNA content ratio analysis

The cells were collected using trypsin/EDTA, and then split into two equal aliquots for protein and DNA measurements. One aliquot was resuspended in protein lysis buffer (50 mmol/L Tris–HCl, pH 7.4, 2 mmol/L EDTA, 1% Nonidet P-40, 100 mmol/L NaCl, 100 mmol/L sodium orthovanadate, and 0.1 mmol/L PMSF with Complete protease cocktail (Roche, Meylan, France)). The protein concentration was determined using a BCA protein assay (Pierce, Rockford, IL, USA). The other aliquot was resuspended in TEN (10 mmol/L Tris–HCl, pH 7.4, 1 mmol/L EDTA pH 8.0, and 150 mmol/L NaCl). The DNA concentration was determined using Hoechst 33258 (Sigma-Aldrich, St. Louis, MO, USA) with calf thymus DNA as a standard (Sigma-Aldrich, St. Louis, MO, USA), as previously described [10]. The DNA was analyzed at 365 nm excitation and 460 nm emission using a fluorescence spectrometer (F-4500, Hitachi, Tokyo, Japan). For each well, the result of protein determination was divided by the DNA measurement to provide a protein/DNA ratio.

### Measurement of fetal gene expression

Cells grown on 30 mm Petri dishes were harvested by scraping. The total RNA was isolated using Trizol (Invitrogen, Carlsbad, CA, USA), according to the manufacturer's instructions, and the concentration was determined using a BioPhotometer (Eppendorf, Hamburg, Germany). Complementary DNA was synthesized from 2.5 μg of total RNA using a PrimeScript RT reagent Kit (TaKaRa, Dalian, China). Real time PCR was performed in triplicate and detected by SYBR Premix Ex Taq™ Kit (TaKaRa, Dalian, China) using the LightCycler2.0 System (Roche, Basel, Switzerland). The relative mRNA expression levels were normalized to 18 s ribosomal RNA. The primers were designed by the web based program, Oligo Perfect™ Designer (Invitrogen). The primers used were as follows: 18 s rRNA forward, 5'-ACGGACCAGAGCGAAAGCAT-3'; 18 s rRNA reverse, 5'-TGTCAATCCT-GTCCGTGTCC-3'; α-MHC forward, 5'-GACACCAGCGCCCACCTG-3'; α-MHC reverse, 5'-ATAGCAACAGCGAGGCTCTTTCTG-3'; β-MHC forward, 5'-GGAGCTCACCTACCAGACAGA-3'; and β-MHC reverse, 5'-CTCAGGGCTTC-ACAGGCATCC-3'.

### Ca^2+^-Transient analysis

Free cytosolic calcium was measured in neonatal rat cardiomyocytes as previously described [11]. Cardiomyocytes were plated onto glass coverslips and cultured for 72 hours. Myocytes were loaded with fura-2/AM (Molecular Probes, Eugen, OR, USA) by incubating the coverslips for 30 minutes in 2 mL of Tyrode’s solution containing (mmol/L) NaCl 137, KCl 5, glucose 15, MgSO_4_ 1.3, NaH_2_PO_4_ 1.2, HEPES 20, and CaCl_2_ 1, as well as fura-2/AM (3 mmol/L) and D-Pluronic (Molecular Probes, Eugen, OR, USA) (3 mL of 25% [wt/wt] in dimethyl sulfoxide). Myocytes were then rinsed with Tyrode’s solution and maintained for 30 minutes at room temperature to allow for deesterification of the dye. Coverslips were transferred to a temperature-regulated chamber (RC-21BRFS, Warner, Hamden, CT, USA) mounted on a Carl Zeiss Axio Observer. A1 microscope, Lambda DG-4 monochromator and filter sets(Sutter, Novato, CA, USA), and cells were perfused with prewarmed modified Tyrode’s solution. Cells were paced by electrical field stimulation (SIU-102, Warner, Hamden, CT, USA) at 1 Hz, 18 V, 4-millisecond pulse duration using platinum electrodes. Fluorescence of intracellular fura-2 was determined by alternatively illuminating cells with 340- and 380-nm light and measuring emission at 520 nm using an EMCCD (iXon Ultra 897, Andor, Belfast, BT, UK). The sampling rate for collection of ratio values was 100 Hz. Images were recorded and analyzed using the MetaFlour software (Molecular Devices, Sunnyvale, CA, USA). As previously reported, improper calibration of fura-2 is difficult to exclude because of compartmentalization in loaded cells and differences in spectral properties between cells and buffer solutions [12]. So the fura-2 fluorescence ratio was used as an indicator of free cellular calcium. Diastolic fluorescence ratio, peak systolic fluorescence ratio, and transient decay (τ, ms) were used for analysis of transient amplitude.

### Measurement of SR Ca^2+^-ATPase activity

The SR membranes from cardiac myocytes were prepared using a modified procedure, as previously described [13]. The cardiac myocytes were homogenized in a buffer containing 300 mmol/L sucrose, 1 mmol/L PMSF, and 20 mmol/L PIPES. The homogenates were centrifuged at 500 *g* for 20 min and at 25000 *g* for 60 min to pellet the SR membranes. The SR Ca^2+^-ATPase activity was measured using a modified method, as previously described [14]. Homogenate protein was added to the solution containing 200 mmol/L KCl, 20 mmol/L HEPES, 10 mmol/L MgCl_2_, 3 mmol/L PEP, 0.6 mmol/L NADH, 1 mmol/L EGTA, 7.5 U/ml PK, and 5 U/ml LDH (pH 7.0), 2 μM of the Ca^2+^ ionophore A-23187 (Sigma-Aldrich, St. Louis, MO, USA). NADH was measured using a photometer (DU-640, Beckman Instruments, Inc., Fullerton, CA, USA) with a wavelength of 340 nm at 37°C, and the reaction was started with 1 mmol/L Na_2_ATP. Absorbance changes were monitored for 3 min. The Ca^2+^ ionophore was added to prevent intraluminal Ca^2+^ accumulation in SR, which is inhibitory to Ca^2+^-ATPase activity [15]. All experiments were performed in triplicate. SERCA2 activity was calculated in nanomoles of ATP per milligram of protein per minute from the change of absorption at 340 nm divided by the extinction coefficient of NADH, in milligrams of protein per minute. Basal activity was measured in Ca^2+^-free buffer in the presence of EGTA (4 mmol/L), followed by increasing Ca^2+^ concentrations.

### Western blotting

The cells were rinsed with cold PBS and then scraped in ice-cold lysis buffer with protease and phosphatase Inhibitor Cocktail (Roche, Meylan, France). The protein concentration was assessed using a BCA Protein assay (Pierce, Rockford, IL, USA). Then, an equal amount of protein was separated using a 4-20% SDS-PAGE and transferred to PVDF membranes (Hybond, Amersham, Arlington Heights, IL, USA). The blots were incubated with the appropriate antibodies, including anti-SERCA2a, anti–phospho-PLN (serine-16, threonine-17) and anti-PLN (Santa Cruz Blotechnology, CA, USA). The signals were detected using Super Signal Immobilon Western Chemiluminescent HRP Substrate (Millipore, France) and quantified by densitometry using the Image analysis system (Bio-Tanon, Shanghai, China). The data were normalized using β-actin to ensure equal loading and were expressed as a ratio of the experimental group to the control group.

### cAMP assay

The concentration of cellular cAMP was carried out using the cAMP-Glo assay kit (Promega, Madison, WI, USA). Briefly, cells were cultured in poly-D-lysine-coated, white, clear-bottom 96-well plates (Greiner, Stonehouse, UK) in a density of 10^4^ cells/well. Cells were lysed to release cAMP, and then the cAMP Detection Solution, which contains protein kinase A (PKA) and kinase substrate (Leu-Arg-Arg-Ala-Ser-Leu-Gly), is added. The Kinase-Glo Reagent is next added to terminate the PKA reaction and detect the remaining ATP via a luciferase reaction. Plates are read using a microplate-reading luminometer (Synergy 2, BioTek, Winooski, VT, US). Relative luminescence output is inversely proportional to cAMP levels.

### PKA assay

Protein kinase A (PKA) activity was performed using the ProFluor PKA assay kit (Promega, Madison, WI, USA). Briefly, cell lysates were added to the reaction buffer containing a bisamide rhodamine 110 peptide substrate and were incubated for 30 min at 25°C. The reaction was stopped by adding the termination buffer, which contained a protease that removes amino acids specifically from the non-phosphorylated substrate and results in the production of highly fluorescent rhodamine 110. Thus, the fluorescence intensity is inversely correlated with kinase activity. PKA activity was determined from the fluorescence intensity of the nonphosphorylated substrate using a Synergy 2 microplate-reader (BioTek, Winooski, VT, US).

### Statistics

Data are presented as the mean ± standard error of mean (SEM). The intergroup variation between various groups was measured by one-way analysis of variance (ANOVA) with Bonferroni’s post hoc test. Results were considered statistically significant when *P* values blow 0.05. Data were analyzed using Statistical Package for Social Science (SPSS, Chicago, IL, USA) software version 14.0 for Microsoft Windows.

## Results

### Effect of STA on NE-induced changes in cell surface area and protein synthesis

**Figure 2:**
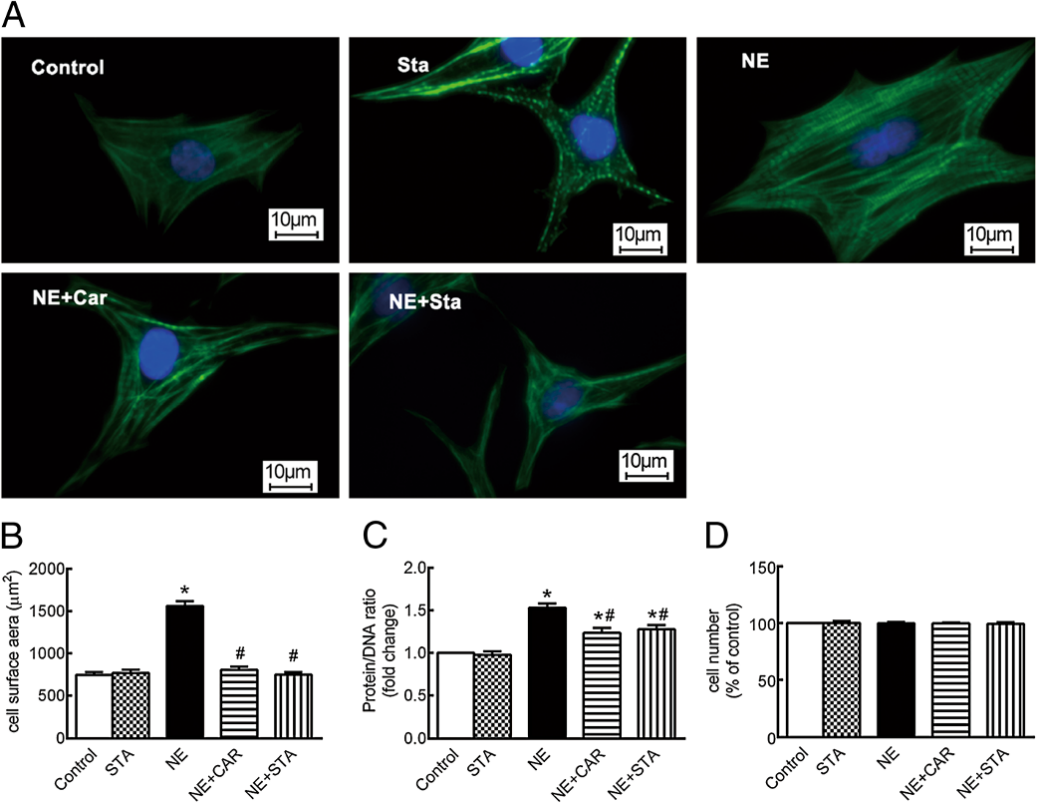
**Effects of STA on cell surface area and protein/DNA ratio changes induced by NE. (A)** Representative fluorescent micrographs of cardiomyocytes; F-actin is visualized with Alexa Fluor 488-conjugated phalloidin stain (green), whereas the nucleus is visualized by DAPI staining (blue), ×400. **(B)** Quantification of cell surface area, n = 150. **(C)** Ratio of Protein/DNA, n = 5. **(D)** Cell viability, n = 4. The data are expressed as the mean ± SEM. *P < 0.05 versus control, ^#^P < 0.05 versus NE. NE: norepinephrine, CAR: carvedilol, STA: stachydrine.

The myocytes treated with NE for 72 hours demonstrated a significant increase in the cell surface area, from 744.74 ± 35.30 μm^2^ to 1606.29 ± 107.91 μm^2^ (Figure 2A and B, *P* < 0.05), whereas in the presence of CAR or STA, the cell surface area in the presence of NE was reduced to 720.35 ± 40.31 μm^2^ and 766.68 ± 14.32 μm^2^, respectively (Figure 2A and B, *P* < 0.05 *vs.* NE group). STA alone had no effects on the cell surface area (Figure 2A and B).

Protein synthesis is evaluated using the ratio of protein/DNA. As shown in Figure 2C, NE induced a 1.51 ± 0.28-fold increase in the protein/DNA ratio compared with the controls. The increase for protein/DNA ratio in the presence of CAR and STA reduced to 1.24 ± 0.16-fold and 1.38 ± 0.22-fold, respectively (*P* < 0.05 *vs.* NE group). STA alone had no effects on the protein/DNA ratio (Figures 2C).

Considering that cell viability may interfere with the protein/DNA ratio, cell viability assays were performed using Cell Counting Kit-8 (CCK-8, Dojindo Molecular Technologies, Kumamoto, Japan), which demonstrated that the cardiac myocyte viability did not significantly change in the presence of NE with or without STA or CAR (Figure 2D).

Together, these results indicate that CAR and STA inhibit the increase in cell surface area and protein synthesis induced by NE.

### Effect of STA on NE-induced changes in the gene expression of β-MHC and α-MHC

**Figure 3:**
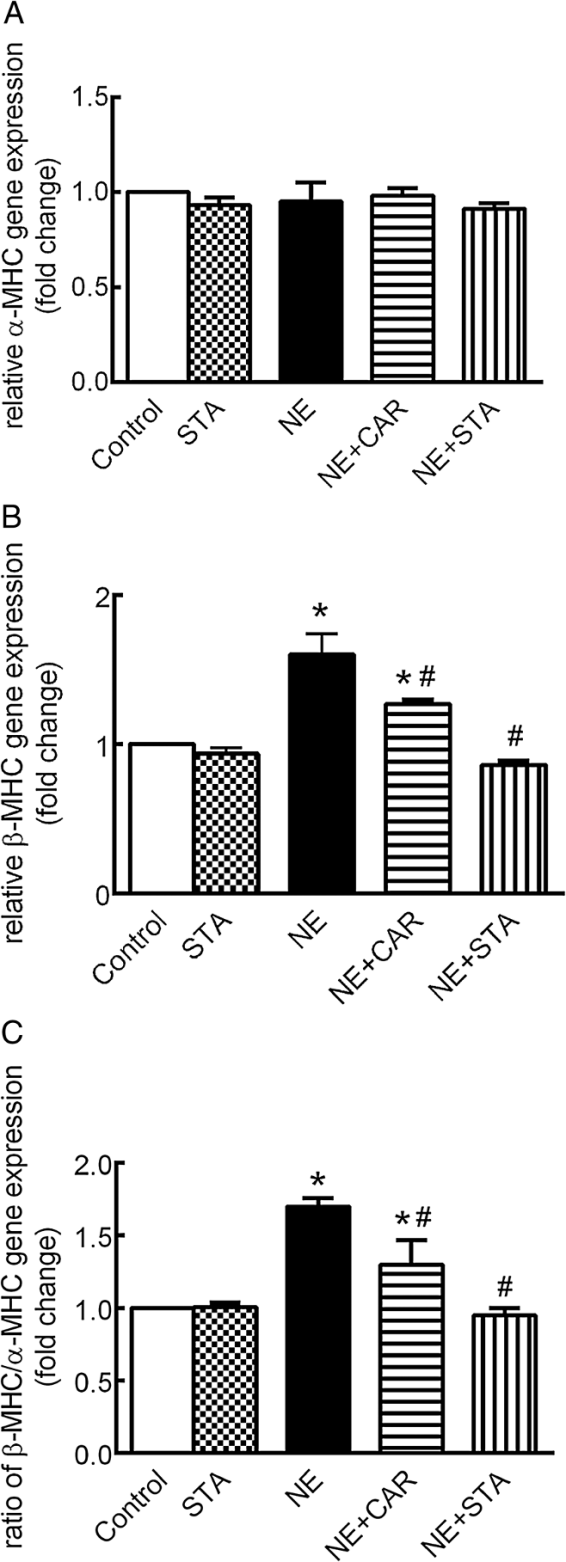
**Effects of STA on NE-induced changes in α- and β-MHC mRNA expression.** qRT-PCR analysis demonstrated **(A)** α-MHC and **(B)** β-MHC gene expression as well as **(C)** β/α-MHC mRNA ratio in the presence of NE with CAR or STA for 72 hours (normalized to 18 s ribosomal RNA). The data are expressed as the mean ± SEM, n = 5. *P < 0.05 versus control, ^#^ *P* < 0.05 versus NE. NE: norepinephrine, CAR: carvedilol, STA: stachydrine. MHC: myosin heavy chain.

Figure 3A illustrates that expression of the α-MHC gene did not change significantly in the presence of NE with or without CAR or STA. However, as shown in Figure 3B, NE induced a 1.60 ± 0.14-fold upregulation of β-MHC gene expression after a 72 hour treatment. The upregulation of the β-MHC gene was abrogated by STA and reduced significantly by CAR. Thereby, the β/α-MHC ratio was increased by NE treatment by 1.70 ± 0.06-fold (Figure 3C) whereas this was completely prevented by STA. While CAR significantly reduced the ratio to 1.297 ± 0.17-fold (Figure 3C, *P* < 0.05 *vs.* NE group). STA alone had no effects on the MHC isoforms gene expression (Figures 3A,B, and C).

### Effect of STA on NE-induced changes in Ca^2+^ transients

**Figure 4:**
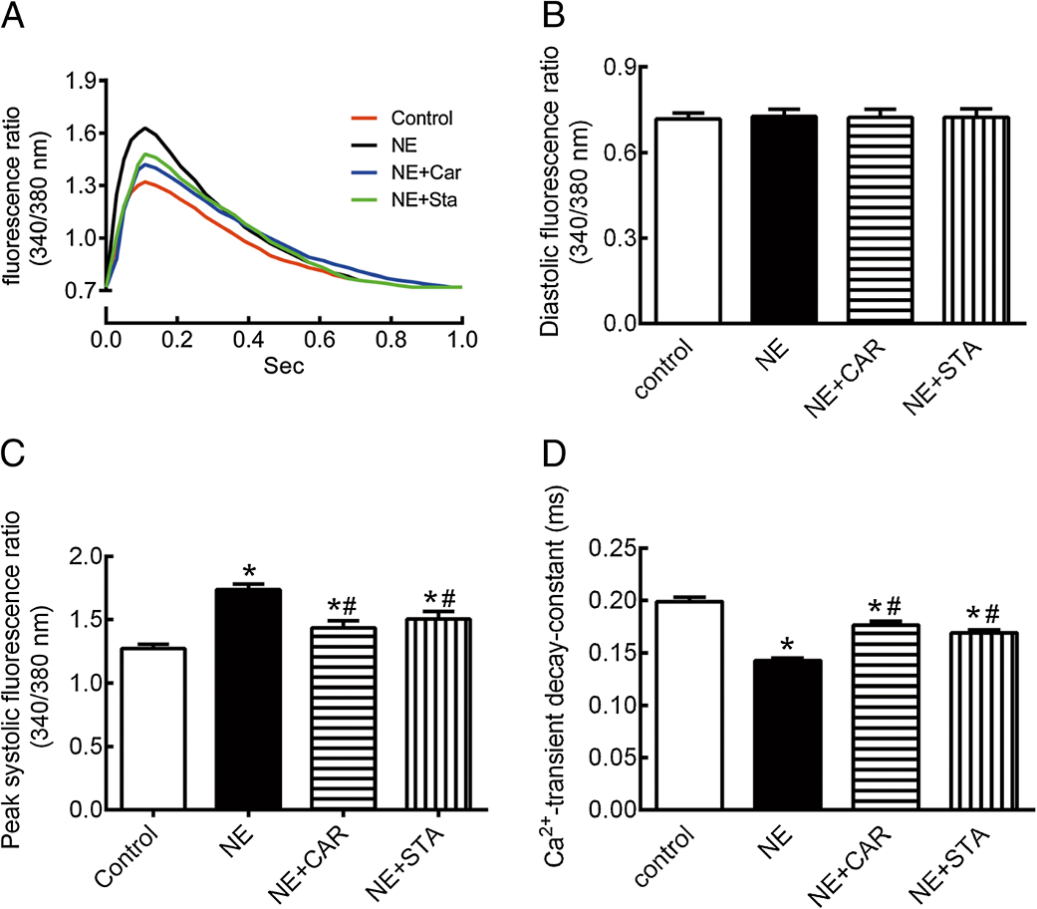
**Effects of STA on Ca** ^**2+**^ **transients changes induced by NE. (A)** Representative calcium transient tracings from cardiac myocytes in NE-treated (NE), NE plus CAR-treated (NE + Car), and NE plus STA-treated (NE + Sta) cells. **(B)** Baseline of diastolic fura-2 ratio. **(C)** Peak systolic fura-2 ratio. **(D)** Ca^2+^-transient decay-constant. These data are expressed as the mean ± SEM. n = 20. *P < 0.05 versus control, ^#^P < 0.05 versus NE. NE: norepinephrine, CAR: carvedilol, STA: stachydrine, SERCA2a: cardiac sarco(endo)plasmic reticulum calcium ATPase.

Figure 4A shows representative calcium transient tracings of a group of normal, NE-treated, CAR-treated, and STA-treated neonatal myocytes. Figure 4B shows that baseline diastolic fura-2 ratio was similar in the four groups. Figure 4C shows that NE significantly enhanced the peak systolic ratio by 36% (*P* < 0.05). The increases for peak systolic ratio in the presence of CAR and STA reduced to 12% and 18%, respectively (*P* < 0.05 *vs.* NE group). Figure 4D shows that the Ca^2+^-transient decay-constant reduced by 28% after NE administration, and steadily prolonged in the presence of CAR or STA (*P* < 0.05 *vs.* NE group).

### Effect of STA on NE-induced changes in SERCA2a activity and protein expression, PLN expression and phosphorylation

**Figure 5:**
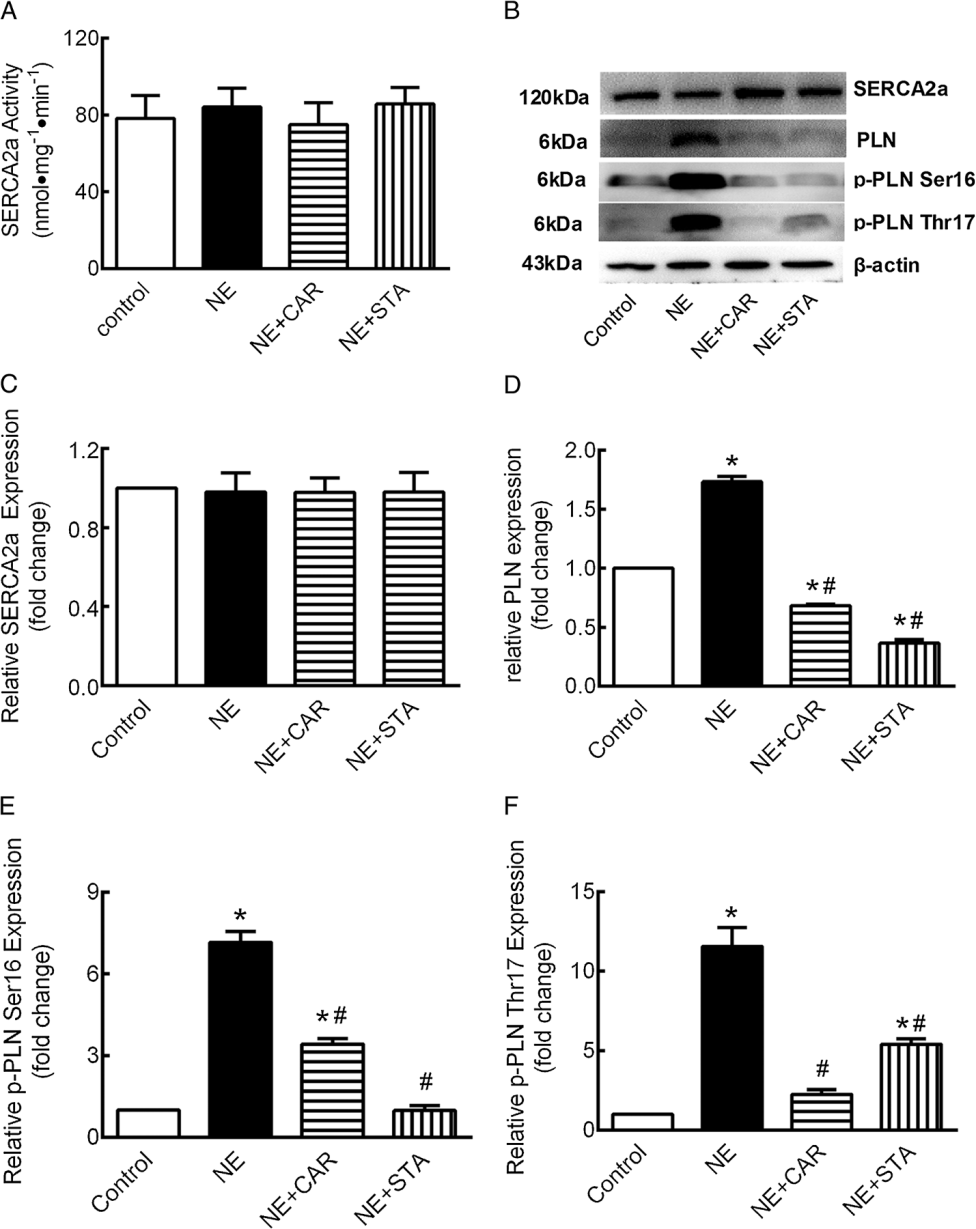
**Effects of STA on SERCA2a and PLN changes induced by NE. (A)** Activity of SERCA2a, **(B)** Representative Western blot, **(C)** quantification of SERCA2a, **(D)** PLN, **(E)** Ser16 phosphorylation, and **(F)** Thr17 phosphorylation. These data were normalized to β-actin. The data are expressed as the mean ± SEM. n = 5. *P < 0.05 versus control, ^#^P < 0.05 versus NE. NE: norepinephrine, CAR: carvedilol, STA: stachydrine, PLN: phospholamban.

Figure 5A and C show that in the presence of NE with or without CAR or STA did not change the activity or expression of SERCA2a significantly.

As shown in Figure 5D, compared with control, PLN protein levels were markedly increased to 1.73 ± 0.10-fold by NE administration for 72 hours (*P* < 0.05). The NE-induced overexpression of PLN protein was significantly decreased by CAR and STA to 0.68 ± 0.03-fold and 0.37 ± 0.07-fold, respectively (*P* < 0.05 *vs.* NE group).

Figure 5E shows that NE dramatically increased PLN phorsphorylation level at serine-16 to 7.161 ± 2.064 fold compared with control (*P* < 0.05); where this was almost completely prevented by STA (*P* < 0.05 *vs.* NE group). While, the abundance of phorsphorylated PLN at serine-16 was also decreased by CAR, although still significantly greater than control.

Similarly, NE significantly increased PLN phorsphorylation level at threonine-17 by 11.56 ± 2.67 fold (Figure 5F, *P* < 0.05) compared with control. CAR and STA decreased the abundance of phorsphorylated PLN at threonine-17 to 2.26 ± 0.68-fold and 5.43 ± 0.80-fold, respectively (*P* < 0.05 *vs.* NE group).

### Effect of STA on NE-induced changes in cAMP content and PKA activation

**Figure 6:**
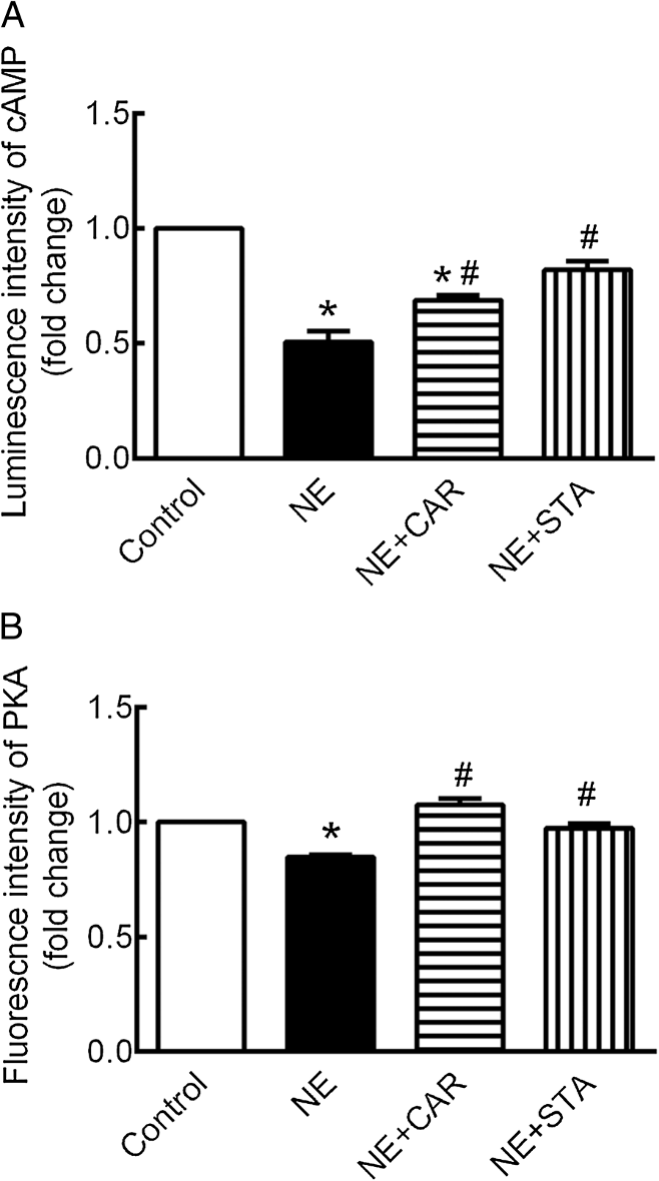
**Effect of STA on cAMP content and PKA activation changes induced by NE.** Quantification of intracellular **(A)** cAMP content and **(B)** PKA activation. The luminescence intensity and fluorescence intensity are inversely correlated with cAMP content and PKA activation. The data are expressed as the mean ± SEM. n = 4. *P < 0.05 versus control, ^#^P < 0.05 versus NE. NE: norepinephrine, CAR: carvedilol, STA: stachydrine, PLN: phospholamban.

Our observation that NE-induced over phosphorylation at serine-16 of PLN almost completely prevented by STA suggests that cAMP/PKA signaling pathway may be involved in the pharmacological profile. To this end, we examined intracellular cAMP content and PKA activation. For the measurement of intracellular cAMP content, the luminescence intensity is inversely correlated with cAMP level. As shown in Figure 6A, the luminescence intensity was markedly decreased by NE administration to 0.5 ± 0.08-fold of control. The decreased of luminescence intensity was abrogated by STA. The decline of luminescence intensity was increased in the presence of CRA, although still significantly lower than control.

Similarly, the fluorescence intensity carried out in PKA assay is inversely correlated with PKA activation. As shown in Figure 6B, the fluorescence intensity was markedly decreased by NE administration to 0.85 ± 0.01 fold of control. The decreased of fluorescence intensity was completely prevented either by CAR or by STA.

## Discussion

The present study determines the potential anti-hypertrophic effects of STA on neonatal rat cardiomyocytes exposed to norepinephrine(NE). Our data demonstrate that STA markedly improve Ca^2+^ handling by reduce Ca^2+^-transient amplitude and prolong Ca^2+^-transient decay constant. Moreover, this study indicates that STA inhibits β1-adrenergic receptors activation, including reduced cAMP content and PKA activation.

Cardiomyocytes response to hypertrophic stimuli by increasing cell size and enhancing protein synthesis [16]. In this study, we demonstrated cell size and protein synthesis dramatically increased by NE treatment, while STA attenuated these changes.

Another feature of cardiomyocyte hypertrophy is reactivation of the fetal cardiac gene program, which includes isoform changes in myosin heavy chain (MHC) [1]. Decrease in fast-shortening-velocity isoform (α-MHC) coupled with increases low-shortening-velocity isoform (β-MHC) may contribute to decreased contractile function [17]. We found that β-MHC mRNA expression increased whereas α-MHC mRNA did not changed significantly in NE induced cardiac hypertrophy. This observation is consistent with other publications [18]. We found STA significantly inhibited the transcription of β-MHC and decreased the β/α-MHC mRNA ratio. Together with data on cell size and protein synthesis, we demonstrated that STA attenuated cardiomyocyte hypertrophy induced by NE.

During cardiac hypertrophy and/or heart failure processes, there is sustained heightened activation of the sympathetic nervous system [19]. Stimulation of β1-adrenergic receptors (β1-AR) by norepinephrine on cardiac myocytes leads to activation of adenylyl cyclases and generation of the cAMP [20]. The primary target for cAMP is protein kinase A (PKA). PKA phosphorylates several proteins that are essential for cardiac function, such as: L-type calcium channels, phospholamban, ryanodine receptors [21]. The most critical functional substrate of the cardiac β-adrenergic pathway is phospholamban (PLN). When PLN is phosphorylated, it released from SERCA2a thus increasing Ca^2+^ pump activity [22].

Acute activation of β1-AR enhances cardiac systolic and diastolic function. However, chronic exposure of the heart to elevated levels of catecholamines may lead to a progressive deterioration in cardiac structure and function. Widely established benefits of β-AR antagonist drugs in treating heart failure strongly support that altered β-AR signaling is maladaptive and promotes heart failure progression [23]. Our data demonstrated that STA attenuated the enhancement of cAMP level and PKA activation induced by NE treatment. Together with the ability to prevent phospholamban phosphorylation at Ser16 indicate that STA may exert antagonistic action at beta-adrenoceptors.

As with all in vitro models, the current results should be interpreted within the constraints of limitations and may not be directly comparable with those obtained in vivo. One of other limitations is the differences in intracellular calcium handling among neonatal and adult myocytes. During relaxation, the prominent mechanisms involved in the decline of intracellular Ca^2+^ concentration are the SERCA2a and the sarcolemmal Na^+^/Ca^2+^ exchanger. Several studies have indicated that the function of SERCA2a in neonatal cardiomyocyte is relatively less important than that in adults [24]. Considering of this, further investigations are needed to clarify the effects of STA on intracellular calcium handling in adult cardiomyocytes.

## Conclusion

This study demonstrated an anti-hypertrophic effect of STA in cultured neonatal rat cardiomyoctes exposed to NE. Our data also indicated that STA has potential cardioprotective effects against β-adrenergic receptor induced Ca^2+^ mishandling.

## Abbreviations

BrdU: 5-bromo-2-deoxy-uridine
CAR: Carvedilol
cAMP: Cyclic adenosine monophosphate
DAPI: 4′, 6′-diamidino-2-phenylindole
DMEM: Dulbecco’s Modification of Eagle’s Medium
EGTA: Ethylene glycol tetraacetic acid
HEPES: 4-(2-hydroxyethyl)-1-piperazineethanesulfonic acid
LDH: Lactate dehydrogenase
NE: Norepinephrine
PEP: Phosphoenolpyruvate
PIPES: Piperazine-1,4-bisethanesulfonic acid
PK: Pyruvate kinase
PKA: Protein kinase A
PLN: Phospholamban
PMSF: Phenylmethylsulfonyl fluoride
SERCA2a: Cardiac sarcoplasmic reticulum calcium ATPase
SR: Sarcoplasmic reticulum
STA: Stachydrine.
